# Safety and efficacy profile of lenvatinib in cancer therapy: a systematic review and meta-analysis

**DOI:** 10.18632/oncotarget.10019

**Published:** 2016-06-14

**Authors:** Chenjing Zhu, Xuelei Ma, Yuanyuan Hu, Linghong Guo, Bo Chen, Kai Shen, Yue Xiao

**Affiliations:** ^1^ State Key Laboratory of Biotherapy and Cancer Center, West China Hospital, Sichuan University, and Collaborative Innovation Center for Biotherapy, Chengdu 610041, PR China; ^2^ West China School of Medicine, West China Hospital, Sichuan University, Chengdu 610041, PR China; ^3^ Department of Oncology, The First People's Hospital of Chengdu City, Chengdu 610041, PR China

**Keywords:** safety, efficacy, lenvatinib, cancer, meta-analysis

## Abstract

To systematically review the safety and efficacy of lenvatinib in the treatment of patients, we retrieved all the relevant clinical trials on the adverse events (AEs) and survival outcomes of lenvatinib through PubMed, Medline, Embase, Web of Science and Cochrane Collaboration's Central register of controlled trial. Fourteen eligible studies involving a total of 978 patients were included in our analysis. The most common all-grade AEs observed in patients treated with lenvatinib were hematuria (56.6%), fatigue (52.2%) and decreased appetite (50.5%). The most frequently observed grade ≥3 AEs were thrombocytopenia (25.4%), hypertension (17.7%) and edema peripheral (15.5%). The incidences of both all-grade and high-grade hypertension were significantly increased. Meanwhile, the controlled trial suggested that progression free survival (PFS) was significantly longer in the lenvatinib group than the placebo group. Subgroup analyses showed that mean PFS for renal cell carcinoma was 10.933±1.828 months (95% CI 7.350-14.515, p < 0.001), and that for thyroid cancer was 18.344±0.083 months (95% CI 18.181-18.506, p < 0.001). In conclusion, lenvatinib is an effective agent in thyroid cancer. Early monitoring and effective management of side effects are crucial for the safe use of this drug.

## INTRODUCTION

Angiogenesis is critical for the local invasion and progression of tumor cells [1]. The aberrant formation and proliferation of blood vessels is due to an imbalance in pro- and anti-angiogenic factors, with the first weighing more [2]. Vascular endothelial growth factor (VEGF), fibroblast growth factor (FGF), platelet-derived growth factor (PDGF) and epidermal growth factor (EGF) are several positive regulators of angiogenesis [3]. Over the last decade, multi-targeted tyrosine kinase inhibitors (TKIs) have been developed and approved in clinical oncology practice [4].

Lenvatinib (E7080) is an oral, multi-targeted tyrosine kinase inhibitor of VEGFR, FGFR, PDGFR and RET [5, 6]. With its anti-angiogenic activity, and a direct effect on tumor cells by preventing relevant signaling pathways [6–8], lenvatinib has been observed to have promising effects in clinical trials for thyroid cancer [9, 10]. In February 2015, US FDA has approved lenvatinib for the treatment of locally recurrent or metastatic, progressive, radioactive iodine-refractory differentiated thyroid cancer (RR-DTC) [9].

Lenvatinib has brought clinical benefits for patients, but adverse events (AEs) are inevitable such as hypertension, fatigue, proteinuria, nausea, decreased weight and abdominal pain, which may decrease the quality of life of patients and influence their acceptance of treatment [11, 12]. Therefore, we conducted a meta-analysis to estimate various AEs and clinical benefits of lenvatinib.

## RESULTS

### Literature search results

**Figure 1:**
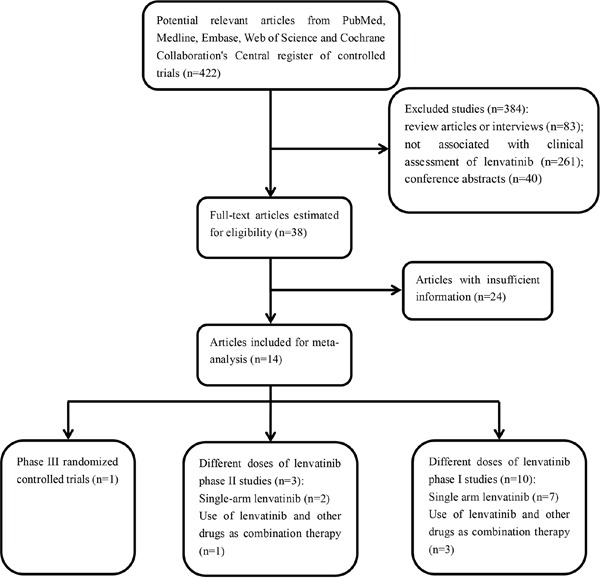
Flow diagram of the literature search and selection process

We ran an initial broad search that yielded 422 unique articles after deletion of duplicates. After title and abstract screening, 344 were excluded since they were narrative review articles or interviews, or completely not associated with clinical assessment of lenvatinib. Forty were further excluded for they were conference abstracts based on published clinical trials, leaving 38 potentially relevant studies for full review. After estimating the full texts of these articles, 24 articles were ruled out for insufficient information. Ultimately, 14 eligible studies [13–26] involving a total of 978 patients met our meta-analysis criteria. Two articles [22, 26] with the same first author which had different study designs were both included in our study, one was a phase II trial, and the other was a phase III, randomized multicenter study. No additional unpublished trials were added to the literature search results. A flow diagram of the trial selection process is provided in Figure 1.

### Study characteristics

Of the studies that were included in the final analysis, 3 studies were based on thyroid cancer patients, 5 evaluated advanced solid tumors, 1 evaluated non-small-cell lung cancer, 1 was based on melanoma, 2 were performed on metastatic renal cell carcinoma, 1 was on advanced hepatocellular carcinoma and 1 on healthy adults. Schlumberger M [22] compared lenvatinib with placebo in radioiodine-refractory thyroid cancer patients, and Motzer RJ [19] used lenvatinib—either in combination with everolimus or as a single agent in patients with metastatic renal cell carcinoma. The characteristics of each trial are summarized in Table 1.

**Table 1:** Basic characteristics of the included articles

First author	Year	Phase	Sample size	Gender		Age	Region	Histology	Treatment arm	Treatment regimen
				Male	Female					
Schlumberger M^1^	2015	II	59	37	22	Mean 52	United States, United Kingdom, Australia, France, Italy, and Poland	MTC or DTC	Lenvatinib	Lenvatinib 24 mg Qd, 28-day cycles
Hong DS^1^	2015	I	77	40	37	Median(range) 61.0(28–85)	USA	Advanced solid tumor; Melanoma	Lenvatinib	Lenvatinib 0.1–3.2 mg Bid (n=18); 3.2–12 mg Bid (n=33); 10 mg Bid (n=26)
Cabanillas ME	2015	II	58	34	24	Median(range) 63(34-77)	USA	RR-DTC	Lenvatinib	Lenvatinib 24 mg Qd, 28-day cycles
Schlumberger M^2^	2015	III	392(lenvatinib: n=261, placebo: n=131)	125, 75	136, 56	Lenvatinib: median 64, placebo: median 61	USA	RR-DTC	Lenvatinib/placebo	Lenvatinib 24 mg Qd, 28-day cycles/placebo
Dubbelman AC	2014	I	6	3	3	Median(range) 49(34–64)	Netherlands	Advanced solid tumors; lymphomas	Lenvatinib	Lenvatinib 24 mg Qd, 28-day cycles
Shumaker RC	2014	I	15	11	4	Median(range) 31(20–49)	USA	Healthy adult	Lenvatinib plus rifampicin	Lenvatinib 24 mg/coadministrate rifampicin 600 mg
Molina AM	2013	Ib	20	14	6	Mean(SD) 58.4(6.29)	Finland	Metastatic renal cell carcinoma	Lenvatinib plus everolimus	Lenvatinib [12 mg (n = 7); 18 mg (n = 11); 24 mg (n = 2)] plus everolimus 5 mg, 28-day cycles
Boss DS	2012	I	82	43	39	Median(range) 54(25–84)	USA	Advanced solid tumours	Lenvatinib	Dose cohorts from 0.2 to 32 mg, 28-day cycles
Nishio M	2013	I	28	21	7	Mean(range) 56.4(38-73)	Japan	Non-small-cell lung cancer	Lenvatinib	Lenvatinib 4/6 mg Bid
Yamada K	2011	I	27	10	17	Median(range) 53(26–70)	Japan	Advanced solid tumours	Lenvatinib	From 0.5 to 1, 2, 4, 6, 9, 13, 16, and 20 mg Bid
Nakamichi S	2015	I	9	2	7	Median(range) 41(30–59)	Japan	Advanced solid tumours	Lenvatinib	Lenvatinib [20 mg (n = 3); 24 mg (n = 6)], 28-day cycles
Hong DS^2^	2015	Ib	32	20	12	Median(range) 57.5(24-81)	USA	Advanced melanoma	Lenvatinib plus TMZ	Dose Level (DL)1: lenvatinib 20 mg, TMZ 100 mg/m2; DL2: lenvatinib 24 mg, TMZ 100 mg/m2; DL3: lenvatinib 24 mg, TMZ 150 mg/m2, 28-day cycles
Ikeda M	2015	I	20	17	3	Median(range) 63.5(47–74)	Japan	Advanced hepatocellular carcinoma	Lenvatinib	Lenvatinib 8 mg, 12 mg, 16 mg, 25 mg Qd, 4-week cycles
Motzer RJ	2015	II	153(lenvatinib: n=52, everolimus: n=50, lenvatinib plus everolimus: n=51)	112	41	Median(range) 59(37–77)	Czech Republic, Poland, Spain, the UK, and the USA	Metastatic renal cell carcinoma	Lenvatinib	Lenvatinib (24 mg/day), everolimus (10 mg/day), or lenvatinib plus everolimus (18 mg/day and 5 mg/day, respectively), 28-day cycles

MTC: medullary thyroid cancer; RR-DTC: radioiodine-refractory, differentiated thyroid cancer

Schlumberger M^1^ and ^2^: the former was a single-arm trial, while the latter was a controlled trial

### Adverse drug reactions analyses

**Figure 2:**
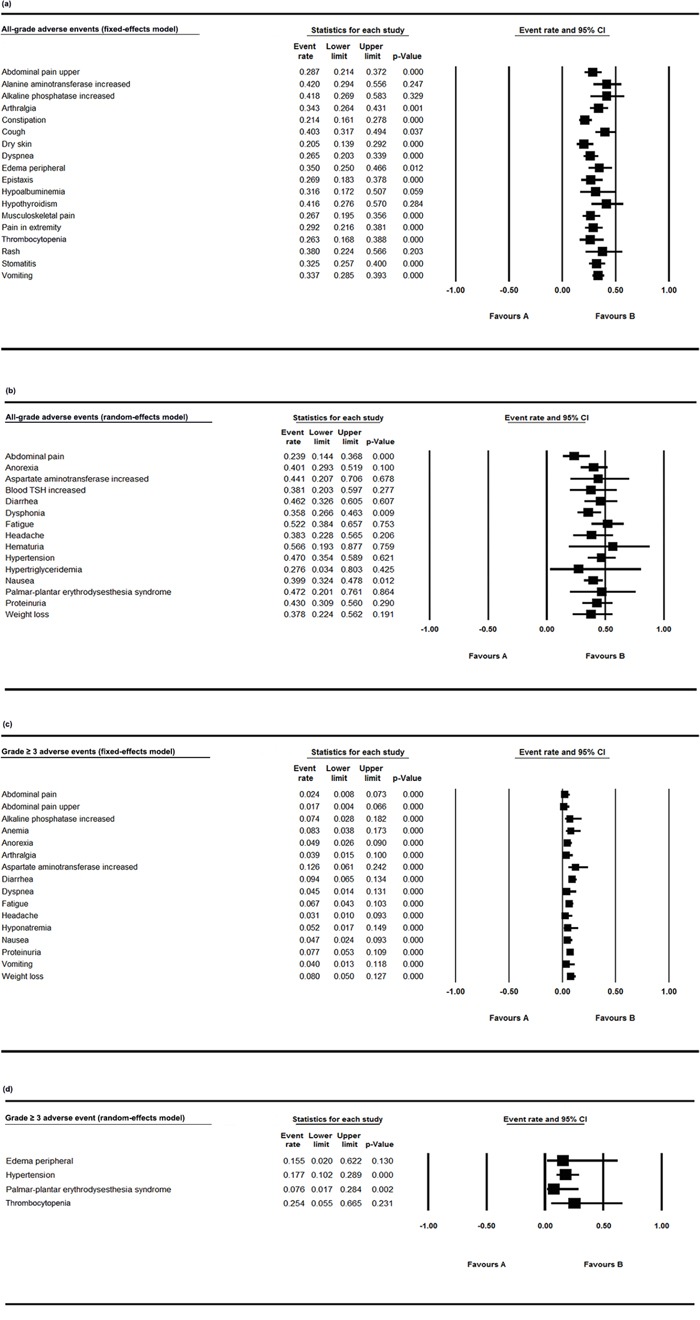
Forest plot of all-grade and grade ≥ 3 AEs in single-arm trials **a.** The all-grade adverse event rates and 95% CIs using a fixed-effects model; **b.** The all-grade adverse event rates and 95% CIs using a random-effects model; **c.** The grade ≥ 3 adverse event rates and 95% CIs using a fixed-effects model; **d.** The grade ≥ 3 adverse event rates and 95% CIs using a random-effects model.

To evaluate the safety of lenvatinib, we calculated the rates of all-grade and grade 3 or more serious adverse events in the overall population. In single-arm trials with all-grade AEs, homogeneity existed in upper abdominal pain, arthralgia, constipation and peripheral edema etc., which were further analysed with a fixed-effects model (Figure 2a, Table 2). Others were analysed using a random-effects model (Figure 2b, Table 2). Hematuria (56.6%, 95% CI 0.193-0.877), fatigue (52.2%, 95% CI 0.384-0.657), palmar-plantar erythrodysesthesia syndrome (47.2%, 95% CI 0.201-0.761), hypertension (47.0%, 95% CI 0.354-0.589) and diarrhea (46.2%, 95% CI 0.362-0.605) were common in a random-effects model (Figure 2b, Table 2). Increased alanine aminotransferase occurred in 42% of the patients using a fixed-effects model (42.0%, 95% CI 0.294-0.556). The most frequent grade ≥ 3 treatment-related adverse events were thrombocytopenia (25.4%, 95% CI 0.055-0.665, random model), hypertension (17.7%, 95% CI 0.102-0.289, random model), peripheral edema (15.5%, 95% CI 0.020-0.622, random model) and increased aspartate aminotransferase (12.6%, 95% CI 0.061-0.242, fixed model) (Figure 2c, 2d, Table 2).

**Table 2:** Summary results of the all-grade and grade ≥ 3 adverse events (AEs) with 95% confidence intervals

All-grade adverse events	Model	Event rate with 95% CI
Abdominal pain upper	Fixed model	0.287 (0.214-0.372)
Alanine aminotransferase increased	Fixed model	0.420 (0.294-0.556)
Alkaline phosphatase increased	Fixed model	0.418 (0.269-0.583)
Arthralgia	Fixed model	0.343 (0.264-0.431)
Constipation	Fixed model	0.214 (0.161-0.278)
Cough	Fixed model	0.403 (0.317-0.494)
Dry skin	Fixed model	0.205 (0.139-0.292)
Dyspnea	Fixed model	0.265 (0.203-0.339)
Edema peripheral	Fixed model	0.350 (0.250-0.466)
Epistaxis	Fixed model	0.269 (0.183-0.378)
Hypoalbuminemia	Fixed model	0.316 (0.172-0.507)
Hypothyroidism	Fixed model	0.416 (0.276-0.570)
Musculoskeletal pain	Fixed model	0.267 (0.195-0.356)
Pain in extremity	Fixed model	0.292 (0.216-0.381)
Thrombocytopenia	Fixed model	0.263 (0.168-0.388)
Rash	Fixed model	0.380 (0.224-0.566)
Stomatitis	Fixed model	0.325 (0.257-0.400)
Vomiting	Fixed model	0.337 (0.285-0.393)
Abdominal pain	Random model	0.239 (0.144-0.368)
Anorexia	Random model	0.401 (0.293-0.519)
Aspartate aminotransferase increased	Random model	0.441 (0.207-0.706)
Blood TSH increased	Random model	0.381 (0.203-0.597)
Diarrhea	Random model	0.462 (0.326-0.605)
Dysphonia	Random model	0.358 (0.266-0.463)
Fatigue	Random model	0.522 (0.384-0.657)
Headache	Random model	0.383 (0.228-0.565)
Hematuria	Random model	0.566 (0.193-0.877)
Hypertension	Random model	0.470 (0.354-0.589)
Hypertriglyceridemia	Random model	0.276 (0.034-0.803)
Nausea	Random model	0.399 (0.324-0.478)
Palmar-plantar erythrodysesthesia syndrome	Random model	0.472 (0.201-0.761)
Proteinuria	Random model	0.430 (0.309-0.560)
Weight loss	Random model	0.378 (0.224-0.562)
**Grade ≥ 3 adverse events**	**Model**	**Event rate with 95% CI**
Abdominal pain	Fixed model	0.024 (0.008-0.073)
Abdominal pain upper	Fixed model	0.017 (0.004-0.066)
Alkaline phosphatase increased	Fixed model	0.074 (0.028-0.182)
Anemia	Fixed model	0.083 (0.038-0.173)
Anorexia	Fixed model	0.049 (0.026-0.090)
Arthralgia	Fixed model	0.039 (0.015-0.100)
Aspartate aminotransferase increased	Fixed model	0.126 (0.061-0.242)
diarrhea	Fixed model	0.094 (0.065-0.134)
Dyspnea	Fixed model	0.045 (0.014-0.131)
Fatigue	Fixed model	0.067 (0.043-0.103)
Headache	Fixed model	0.031 (0.010-0.093)
Hyponatremia	Fixed model	0.052 (0.017-0.149)
Nausea	Fixed model	0.047 (0.024-0.093)
Proteinuria	Fixed model	0.077 (0.053-0.109)
Vomiting	Fixed model	0.040 (0.013-0.118)
Weight loss	Fixed model	0.080 (0.050-0.127)
Edema peripheral	Random model	0.155 (0.020-0.622)
Hypertension	Random model	0.177 (0.102-0.289)
Palmar-plantar erythrodysesthesia syndrome	Random model	0.076 (0.017-0.284)
Thrombocytopenia	Random model	0.254 (0.055-0.665)

### Survival outcomes and subgroup analysis

The efficacy analysis of lenvatinib was mainly based on the controlled trial of lenvatinib in patients with thyroid cancer [22]. The median progression-free survival was 18.3 months in the lenvatinib group and 3.6 months in the placebo group (hazard ratio for progression or death 0.21, 99% CI 0.14-0.31, P < 0.001). In addition, Motzer RJ [19] reported that median PFS was 7.4 months (95% CI 5.6-10.2) for single-agent lenvatinib in patients with metastatic renal cell carcinoma and 5.5 months (95% CI 3.5-7.1) for single-agent everolimus, representing the significantly prolonged PFS of lenvatinib compared with everolimus alone (HR 0.61, 95% CI 0.38-0.98, p = 0.048). Seven trials [13, 15, 16, 19, 22, 25, 26] reported encouraging response rates, median time to response, or PFS observed in patients with different types of tumors, demonstrating the anti-tumour efficacy of lenvatinib (Table 3). We further carried out subgroup analyses according to tumor types. Mean PFS for renal cell carcinoma was 10.933 ± 1.828 months (95% CI 7.350-14.515, p < 0.001), and that for thyroid cancer was 18.344±0.083 months (95% CI 18.181-18.506, p < 0.001) (Table 4). Further large-scale studies are still needed to assess the PFS of patients with melanoma and non-small-cell lung cancer.

**Table 3:** The median PFS of the included trials

Study	Sample size	Tumor types	Median PFS (95%CI) (Months)	Mean	SD	Overall median OS
Boss DS 2012	9	renal cell carcinoma	15.9 (9.3-18.63)	14.93	2.75	
	14	melanoma	7.23 (3.63-12.63)	7.68	2.61	
Schlumberger M^1^ 2015	59	MTC	9.0 (7-16.6)	6.25	2.4	16.6 (16.4-NE)
Cabanillas ME 2015	58	RR-DTC	12.6 (9.9-16.1)	12.8	1.55	
Molina AM 2014	20	renal cell carcinoma	11 (5.23-14.87)	10.525	2.41	
Nishio M 2013	28	non-small-cell lung cancer	9.0 (6.5-9.5)	8.5	0.75	
Schlumberger M^2^ 2015	392	RR-DTC	18.3 (15.2-26)	19.45	1.8	
Motzer RJ 2015	153	renal cell carcinoma	7.4 (5.6-10.2)	7.65	0.77	

SD: Standard deviation estimation

MTC: medullary thyroid cancer

RR-DTC: radioiodine-refractory, differentiated thyroid cancer

Martin schlumberger ^1^ and ^2^: the former was a single-arm trial, while the latter was a controlled trial

**Table 4:** Subgroup analysis for survival outcomes

First author	Model	Mean	Standard error	Variance	95% CI		Z-Value	P-Value	Histology
					lower limit	upper limit			
Cabanillas ME 2015		12.800	0.204	0.041	12.401	13.199	62.892		RR-DTC
Schlumberger M^2^ 2015		19.450	0.091	0.008	19.272	19.628	213.933		RR-DTC
Overall	Random	18.344	0.083	0.007	18.181	18.506	220.987	< 0.001	
Molina AM 2014		10.525	0.539	0.290	9.469	11.581	19.531		RCC
Boss DS 2012		14.930	0.917	0.840	13.133	16.727	16.287		RCC
Motzer RJ 2015		7.650	0.062	0.004	7.528	7.772	122.890		RCC
Overall	Random	10.933	1.828	3.341	7.350	14.515	5.981	< 0.001	

MTC: medullary thyroid cancer

RR-DTC: radioiodine-refractory, differentiated thyroid cancer

RCC: Renal cell carcinoma

Schlumberger M^1^ and ^2^: the former was a single-arm trial, while the latter was a controlled trial

### Risk of bias and quality assessment

**Figure 3:**
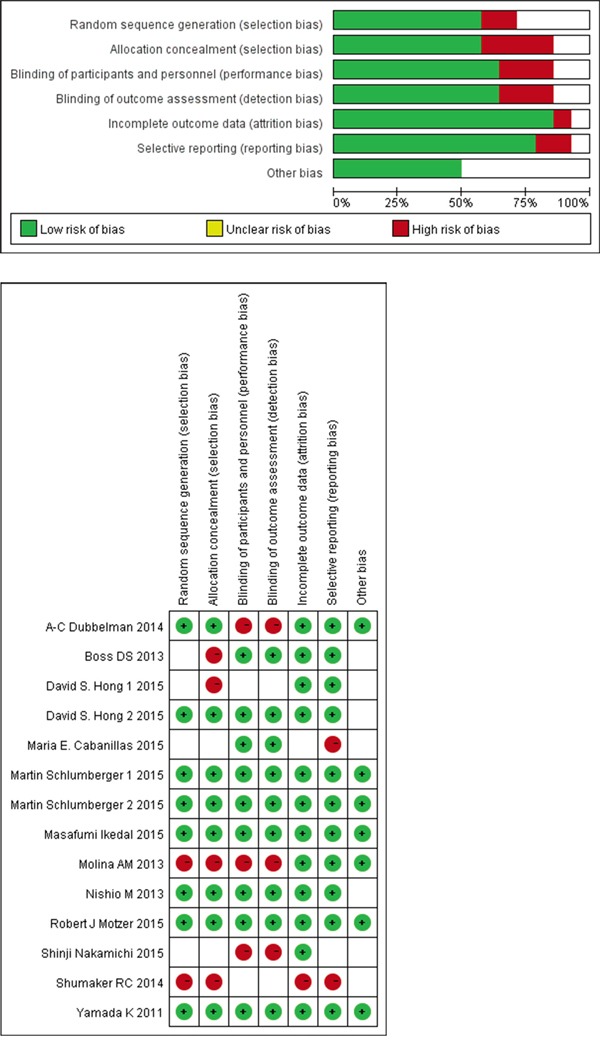
Risk of bias and quality assessment **a.** Risk of bias graph: review authors' judgments about each risk of bias item presented as percentages across all included studies; **b.** Risk of bias summary: review authors' judgments about each risk of bias item for each included study.

The risk of bias and quality assessments of the included studies are outlined in Figure 3a, 3b. Overall, the quality of the studies was satisfactory.

## DISCUSSION

To the best of our knowledge, this is the first study to evaluate both the safety and efficacy of the novel antitumor agent lenvantinib in different types of tumors systematically. The adverse events of lenvatinib were tyrosine kinase inhibitor-related and were also seen in other TKIs. In one meta-analysis [27], the VEGFR-TKIs group (cediranib and axitinib) was associated with higher rates of diarrhea, fatigue, hypertension and thrombocytopenia compared with bevacizumab. Vandetanib [28], a dual VEGFR and EGFR inhibitor, yielded an improvement in PFS but more frequent grade 3 or greater hypertension. Although the incidence of hematuria was high, most people experienced low grade (grade 0) hematuria.

It should be noted that lenvatinib was associated with a significantly increased risk in all-grade (47.0%) and high-grade (17.7%) hypertension. The mechanism of lenvatinib-associated hypertension has not been clarified, and may be due to a possible perturbation of endothelial cell function in patients treated with VEGF-targeting agents [29]. It has been documented upon administration of bevacuzimab and cediranib, and several other inhibitors of the VEGF signalling pathway [30–32]. All of these suggest that patients who were administered lenvatinib should be monitored for high blood pressure, and managed with antihypertensive drugs or dose reductions when necessary.

Grade ≥ 3 thrombocytopenia was experienced in about a quarter of patients. Through binding to PDGFR, PDGF promotes the recovery of platelets and the formation of bone marrow colony-forming unit-megakaryocyte [33, 34], thus the inhibition of PDGFR by lenvatinib might cause thrombocytopenia. Hematopoietic growth factors and transfusions [35] could be used to deal with persistent toxicities on platelets, but the effects of them on tumor cells remain to be explored.

**Figure 4:**
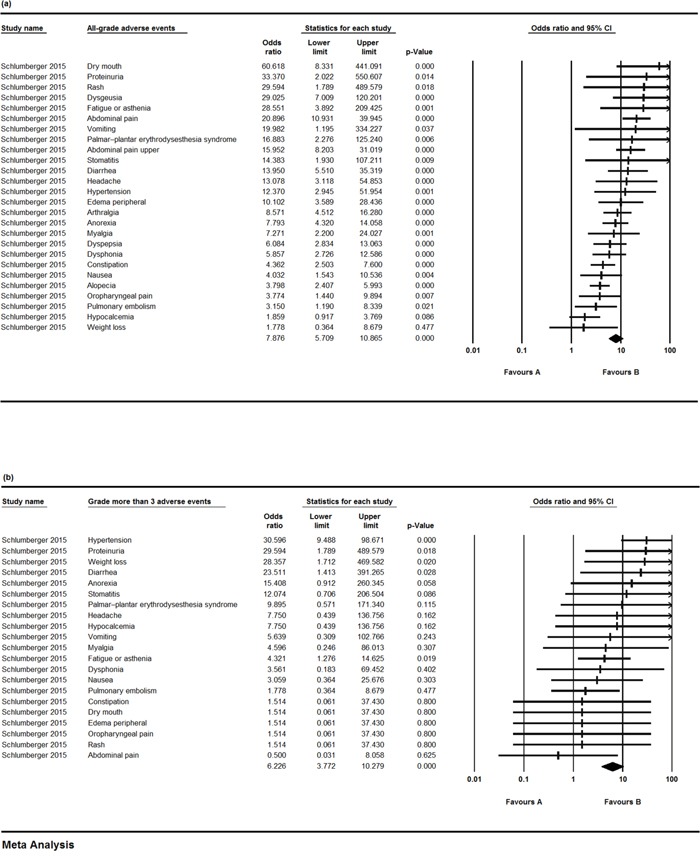
The odds ratios (ORs) of adverse events (AEs) in a controlled trial comparing lenvatinib and placebo **a.** OR and 95% CIs of all-grade AEs using a random-effects model; **b.** OR and 95% CIs of grade ≥ 3 AEs using a fixed-effects model.

In February 2015, US FDA has approved lenvatinib for the treatment of radioiodine-refractory thyroid cancer [9] based on the randomized controlled trial [22] included in our analysis. We find a similar mean PFS (18.344±0.083 months, 95% CI 18.181-18.506, p < 0.001) for thyroid cancer in our pooled analysis. However, our results of adverse events (Figure 4a, 4b) are different, since the relatively larger sample size may allow us to better determine the AE values. Survival outcomes of other tumors are mainly based on phase I and phase II trials, and more subsequent randomized, controlled phase III trials are needed.

The dose of lenvatinib administered in patients with solid tumors varied in different situations, but in 8 [15, 16, 18–20, 22, 23, 26] of the 14 included studies, patients received lenvatinib at a daily dose of 24 mg per day in 28-day cycles, and two studies [18, 20] demonstrated that the 24-mg QD dose of lenvatinib was determined to be tolerable with encouraging anti-tumor activity in patients with solid tumors.

The heterogeneity in our analysis could arise from different tumor types, the very heterogeneous study population with pre-treated disease and the ethnicity difference. In addition, there are several limitations of our study. Firstly, because lenvatinib is a relatively new drug, reports about it are few and are mostly phase I and II studies. Secondly, only one study provided the overall survival data, so prolonged follow-ups are needed. Thirdly, we did not perform subgroup analysis of melanoma and non-small-cell lung cancer because of lack of enough information.

In conclusion, lenvatinib has clinically meaningful benefits in survival outcomes of patients with thyroid cancer. The pooled analyses suggest that patients should be monitored for potential thrombocytopenia and increases in blood pressure, and dose reductions or delays or antihypertensive drugs are needed accordingly. Correct estimates of treatment-related toxicities and the efficacy of lenvatinib are fundamental to provide appropriate guidance and to conduct ongoing trials.

## MATERIALS AND METHODS

### Search strategy

We performed a literature search of PubMed, Medline, Embase, Web of Science and The Cochrane Library for all the relevant clinical trials on the safety and efficacy of lenvatinib (until April 26, 2016, 201). In order to ensure the completeness of the results, we expanded the search scope by using the search terms “lenvatinib” or “E7080” or “lenvima”. We also carried out further searches for relevant unpublished trials in the clinical trial registry (http://www.clinicaltrials.gov). Papers in all languages were sought and translated where appropriate to reduce the chances of bias.

### Inclusion and exclusion criteria

To be included in the analysis, patients must be diagnosed with histologically confirmed tumors, survival outcomes and toxicities were mandatory to be reported. All phase clinical trials were eligible for inclusion if they evaluated the side effects and efficacy of lenvatinib. Studies were excluded if they did not provide enough data for toxicities and survival outcomes. They were also excluded for which full-text reports were not available.

### Selection process and data extraction

Two reviewers selected studies independently. Any disagreements were resolved through discussion with another author. We excluded those studies that clearly did not meet the inclusion criteria, and made efforts to rule out duplicated studies by comparing author lists, publication year, and the main contents if necessary. Articles with the same author(s) or medical center(s) were carefully reviewed and discussed for eligibility.

Data extracted from all eligible articles included the first author, year of publication, sample size, study phase, tumor type, treatment regime, progression-free survival (PFS), hazard ratio (HR) and adverse events. ADRs were graded using the National Cancer Institute (Washington, DC, USA) Common Toxicity Criteria, version 3.0.

### Data analysis

We used patients' all-grade and grade ≥ 3 Common Toxicity Criteria Adverse Events (CTCAE) counts to calculate the incidence rates of AEs and the corresponding 95% confidence intervals (CIs). I-squared was calculated to test heterogeneity of the studies, and I^2^ > 50% and P ≤ 0.1 indicated strong heterogeneity between the studies. All the analysis was carried out using the software Comprehensive Meta-Analysis (CMA) program 2 (Biostat, Englewood, NJ) and Review manager 5.3 (Copenhagen, Sweden).

### Risk of bias and quality assessment

To evaluate the risk of bias and quality of the studies, QUADAS-2 was used as a systematic review assessment method, which consisted of four key domains: patient selection, index test, reference standard and flow and timing [36]. Risk of bias was rated as high/low/unclear. The assessment was measured using Review Manager 5.3 (Copenhagen, Sweden).
